# Metropolitan and Central Medical Ethics

**Published:** 1860-07

**Authors:** 


**Metropolitan and Central Medical Ethics.**

We perceive through the *Newspapers* that certain Surgical operations have been recently successfully performed in *New York* and *Philadelphia* by certain prominent Surgeons connected with the leading schools of those cities. With such facts as these and the regular publication of the proceedings of the New York Academy of Medicine through the same channel, who shall gainsay the right of any medical man to set up for himself and herald his own feats in any manner best suited to advance his own interest. The New York Medical Gazette, then notices the New York performance:

Advertising Doctors.—This tribe, so long denounced, must be taken into favor again. Several of the journals are complaining hugely that young doctors are not allowed to advertise themselves and their cures, while the old ones, after having made their fortunes in this way, continue to puff themselves in the newspapers, by some ingenious device. A professor and hospital surgeon, in New York, lately had a lucky case of Tracheotomy in Croup, which is now going the rounds of penny and Sunday press. He is careful not to tell how many times he and others have lost children in this way. Nor does he confess the truth that this child might have got well without having its throat cut, so that it may have been an extraordinary escape, not a cure.

But the advertisement answers his purpose, which is to invite calls to his shop in cases of croup. The statistics of the mortality in croup after this operation, prove that there would be more recoveries, if the patients were let alone.—But if he is thus to advertise, why should not all the young doctors do so with impunity? Circumstances alter cases. *Vive la bagatelle.*

